# Mutant p53 Gain-of-Function Induces Migration and Invasion through Overexpression of miR-182-5p in Cancer Cells

**DOI:** 10.3390/cells12202506

**Published:** 2023-10-23

**Authors:** Tzitzijanik Madrigal, Daniel Ortega-Bernal, Luis A. Herrera, Claudia Haydée González-De la Rosa, Guadalupe Domínguez-Gómez, Elena Aréchaga-Ocampo, José Díaz-Chávez

**Affiliations:** 1 Unidad de Investigación en Cáncer, Instituto de Investigaciones Biomédicas-Universidad Nacional Autónoma de México, Instituto Nacional de Cancerología, San Fernando 22, Sección XVI, Tlalpan, CDMX, Mexico City 14080, Mexico; tzitzita@hotmail.com (T.M.); metil@hotmail.com (L.A.H.); 2 Departamento de Ciencias Biológicas y de la Salud, UAM Iztapalapa, Mexico City 09340, Mexico; 3 Departamento de Atención a la Salud, UAM Xochimilco, Mexico City 04960, Mexico; tse_lao_do@yahoo.com.mx; 4 Departamento de Ciencias Naturales, Unidad Cuajimalpa, Universidad Autonóma Metropolitana, Mexico City 05348, Mexico; cgonzalez@cua.uam.mx (C.H.G.-D.l.R.); earechaga@cua.uam.mx (E.A.-O.); 5 Escuela de Medicina y Ciencias de la Salud-Tecnológico de Monterrey, Mexico City 14380, Mexico; 6 Subdirección de Investigación Clínica, Instituto Nacional de Cancerología, Mexico City 14080, Mexico; dguadalupeisabel@yahoo.com.mx

**Keywords:** miRNAs, mutant p53, gain of function, cancer

## Abstract

The master-key TP53 gene is a tumor suppressor that is mutated in more than 50% of human cancers. Some p53 mutants lose their tumor suppressor activity and acquire new oncogenic functions, known as a gain of function (GOF). Recent studies have shown that p53 mutants can exert oncogenic effects through specific miRNAs. We identified the differentially expressed miRNA profiles of the three most frequent p53 mutants (p53R273C, p53R248Q, and p53R175H) after their transfection into the Saos-2 cell line (null p53) as compared with p53WT transfected cells. The associations between these miRNAs and the signaling pathways in which they might participate were identified with miRPath Software V3.0. QRT-PCR was employed to validate the miRNA profiles. We observed that p53 mutants have an overall negative effect on miRNA expression. In the global expression profile of the human miRNome regulated by the p53R273C mutant, 72 miRNAs were underexpressed and 35 overexpressed; in the p53R175H miRNAs profile, our results showed the downregulation of 93 and upregulation of 10 miRNAs; and in the miRNAs expression profile regulated by the p53R248Q mutant, we found 167 decreased and 6 increased miRNAs compared with p53WT. However, we found overexpression of some miRNAs, like miR-182-5p, in association with processes such as cell migration and invasion. In addition, we explored whether the induction of cell migration and invasion by the p53R48Q mutant was dependent on miR-182-5p because we found overexpression of miR-182-5p, which is associated with processes such as cell migration and invasion. Inhibition of mutant p53R248Q and miR-182-5p increased FOXF2-MTSS1 levels and decreased cell migration and invasion. In summary, our results suggest that p53 mutants increase the expression of miR-182-5p, and this miRNA is necessary for the p53R248Q mutant to induce cell migration and invasion in a cancer cell model.

## 1. Introduction

p53WT is a tumor suppressor protein encoded by the TP53 gene located on chromosome 17p13.1. In response to cellular stress, p53 activates the expression of several genes associated with cell cycle arrest, apoptosis, and DNA repair; however, p53 is mutated in more than 50% of human cancers [1]. About a third of these missense mutations are located in six residues: the R175, G245, R248, R249, R273, and R282 hot spots. These six residues account for 30% of p53 gene mutations in all human cancers. The codons R175, R248, and R273 are more frequently mutated than the others [2,3]. 

TP53 missense mutations occur mainly in the DNA-binding domain and can be classified into two main categories. These two categories of mutations are commonly referred to as conformational or DNA contact mutations, or class I or II, respectively [4]. Class I involves the substitution of an amino acid residue that causes loss of contact with DNA, affecting its transcriptional capacity; within this category are mutations at positions R248Q and R273C. Mutated class II proteins present structural changes (R175H) that affect their affinity for DNA. Class I mutants of p53 usually have a native conformation, whereas class II are unable to acquire the native conformation and, therefore, misfold [5]. 

The effects of TP53 mutations can be classified into three groups. First, p53 mutations attenuate binding to its DNA response elements and block transcriptional activation of p53 target genes, so a partial or total loss function can define these mutations. Second, p53 mutant proteins exert a dominant-negative effect on the function of wild-type (WT) p53 protein, encoded by the second allele, through the formation of a heterotetramer deficient in its binding to specific DNA sequences, also known as dominant-negative mutations. Finally, some p53 mutants also acquire new functions independent of p53WT; this event is known as a gain of function (GOF). GOF p53 mutations are involved in critical oncogenic processes, such as increased cell migration and invasiveness [6,7]. 

The effects of TP53 mutations on function and cellular behavior depend on the cell type and environmental conditions [8]. Thus, mutant p53 proteins are able to interact with specific intracellular proteins and induce gene expression changes [9,10]. Moreover, GOF activities of p53 missense mutations vary depending on the mutation type, giving rise to phenotypic differences in vivo associated with the development of different cancer types [11]. In summary, understanding how p53 mutants induce phenotypic differences may help cancer prevention and therapy strategies.

In the present study, we explored the possibility that p53 mutant proteins exert their gain-of-function activity by modulating the expression of miRNAs. miRNAs are 20–24 nucleotides in length and are involved in the post-transcriptional control of genes [12]. Recently, it has been reported that some miRNAs also play an important role in the gain of function of mutant p53; it has been demonstrated how p53 mutants regulate gene expression and exert oncogenic effects by unbalancing specific microRNA (miRNA) levels and even disrupting their biogenesis, which provokes epithelial–mesenchymal transition, chemoresistance, and increased cell survival, among other effects [13,14]. However, the details of how and which miRNAs are regulated by p53 mutants, promoting tumorigenesis, are not yet fully understood. 

In this study, we demonstrated that the three most frequent p53 mutants in cancer induce overexpression of miR-182-5p; moreover, miR-182-5p overexpression is required for the p53R248Q mutant to induce cell invasion and migration in Saos-2 and OVCAR-3 cell lines.

## 2. Materials and Methods

### 2.1. Cell Culture and Treatments

We selected the Saos-2/Osteosarcoma cell line, devoid of any endogenous p53 gene. In addition, SKBR3/Breast carcinoma (p53R175H), C33a/Cervical cancer (p53R273C), and OVCAR-3/Ovarian cancer (p53R248Q) were used because they have the most frequent endogenous p53 mutants. All of these cell lines were cultured in DMEM medium (Gibco, Grand Island, NY, USA) supplemented with 10% fetal bovine serum (Gibco). The non-tumorigenic breast epithelial cell line MCF10a (p53WT) was cultured in DMEM-F12 medium (Gibco) supplemented with 10% SFB, 10 μg/mL insulin (Gibco), 0.5 μg/mL hydrocortisone (Sigma-Aldrich, St. Louis, MO, USA), and 20 ng/mL recombinant human epidermal growth factor (Gibco). All cells were obtained from the American Type Cell Culture Collection (ATCC, Manassas, VA, USA) and were maintained at 37 °C and 5% CO_2_. 

For treatments with pifithrin-α hydrobromide (CAS 63208-82-2, Santa Cruz, Santa Cruz, CA, USA, cat # sc-126 HRP), OVCAR-3 cells were treated with or without the drug (30, 50, 75, or 100 μM) for 24 h. The vehicle was dimethyl sulfoxide (DMSO). Subsequently, the cells were collected and used in the corresponding experiments.

### 2.2. Plasmids and Transfections

Saos-2 cells at 70% confluence were transfected with p53R273C [15], p53R175H [16], p53R248Q [15], p53WT [17], or empty vector. Transfections corresponding to p53 mutants (R273C, R175H, or R248Q) and wild type (p53WT) were performed using Lipofectamine 3000 Reagent (Invitrogen, Carlsbad, CA, USA) according to the manufacturer’s instructions. Clone selection for transfections was performed using 800 μg/mL G418 (Sigma-Aldrich, St. Louis, MO, USA). 

The activity of miR-182-5p was inhibited using the Anti-miR™ miRNA Inhibitor (MH12369) Ambion^®^, Carlsbad, CA, USA. For this purpose, Saos-2 cells were transfected with 10 μM negative control #1 (AM17010), and Saos-2 (p53R248Q) cells were transfected with 10 μM of the negative control and/or anti-miR. This was done using Lipofectamine 3000 following the manufacturing instructions (Invitrogen). Samples were collected within 24 h for further experiments.

### 2.3. RNA Extraction

Total RNA was extracted with the TRIzol method (Invitrogen, USA) according to the protocol provided by the manufacturer. The RNA concentration was measured using a NanoDrop ND-2000 (Thermo Scientific, Waltham, MA, USA), and the RNA quality was evaluated with a Tape Station 2200 bioanalyzer (Agilent Technologies, Santa Clara, CA, USA), with minimum quality requirements: A260/280 ≥ 1.8; RNA integrity number (RIN) ≥ 7.

### 2.4. Reverse Transcription

Using the Applied Biosystems miScript RT II kit, cDNA was synthesized following the manufacturer’s recommendations. The RT reaction was performed with a GeneAmp System 7500 thermal cycler (Applied Biosystems, Waltham, MA, USA). 

### 2.5. Human miRNome PCR Array

Expression profiling was performed with miScript miRNA PCRNA Arrays Human miRNome (384-well plate) (MIHS-3216Z) QIAGEN, which is based on 1066 miRNAs reported in miRbase Release 16 (www.miRBase.org (accessed on 25 June 2020), plus controls. SYBR Green qPCR was performed as follows: 2.5 μL of the cDNA (p53WT, p53R275H, p53R248Q, or p53R273C) were mixed with 5 μL of RNAse-free water plus 12.5 μL of 2x QuantiTect SYBR Green PCR Master Mix and 2.5 μL of 10x miScript Universal primer. The reaction was carried out by programming one step at 95 °C for 15 min, followed by 40 cycles of three temperatures 94 °C for 15 s, 55 °C for 30 s, and 70 °C for 30 s. The amplification reactions were completed using the QuantStudio 6 Flex Real-Time PCR System (Life Technologies, Carlsbad, CA, USA).

### 2.6. PCR Array Analysis

The data were analyzed with “Web-based miScript miRNA PCR Array data analysis tool”, which allowed us to generate a list of differentially expressed miRNAs using the comparative CT (2^−∆∆Ct^) method. The expression level in the cell line transfected with p53WT was considered a calibrator, and the expression levels of the small nucleolar RNAs RNU6 and RNU48 were considered normalizers. Differentially expressed miRNAs were considered to be those with a rate of change value of ≥2 or ≤−2 concerning the control and presenting statistical confidence or a *p*-value < 0.05.

### 2.7. Bioinformatic Analysis and Visualization

All differentially expressed miRNAs from the three comparison analyses performed (p53R273C vs. p53WT, p53R248Q vs. p53WT, and p53R175H vs. p53WT) were used. We applied a heatmap with an unsupervised hierarchical clustering approach with the gplots 2.14.1 library [18] of the Bioconductor library [19] of the R 3.1.1 statistical software [20].

Differential expression visualization was performed using Circos plots with the Circos tool [21]. To identify similarities of differentially expressed miRNAs between comparisons, the online tool Venny 2.1 was used [22].

Enrichment results of the differentially expressed miRNAs were processed through the online software miRPath 3.0 [23]. The microT-CDS 5.0 algorithm was used to determine hypothetical target genes, and DIANA-TarBase 7.0 was used for experimentally validated targets. To identify signaling pathways altered by miRNAs, information from the Kyoto Encyclopedia of Genes and Genomes (KEGG) pathways was used [24]. Pathways were considered altered when *p* < 0.05, and they were visualized by dotplots constructed with R 3.1.1 statistical software.

### 2.8. Validation of miRNA Expression by RT-qPCR

Taqman probes specific for hsa-miR-509-5p (ID: 002235), hsa-miR-3151 (ID: 243597 MAT), has-miR-27b (ID 002174), has-miR-200c* (ID: 002286), has-miR-517a (ID: 001151), has-miR-101 (ID: 002253), miR-885-3p (ID 002372) and hsa-miR-182 (ID: 002334), as well as the internal control RNU6 (ID: 001093), were purchased from Ambion (Applied Biosystems, Foster City, CA, USA) (P/N: 4427975). First, miRNA RT was performed using stem–loop primers (Applied Biosystems). For this, 5 μL (100 ng/μL) of total RNA was added to a mixture containing: 0.15 μL of dNTPs with dTTP (100 mM), 3 μL of miRNA RT primers, 1.0 μL of MultiScribe reverse transcriptase enzyme (50 U/μL), 1.5 μL of 10X RT buffer, 0.19 μL of RNase inhibitor (20 U/μL), and 4.16 μL of RNase-free water, totaling 15 μL per reaction. The RT reaction was performed on a GeneAmp System 9700 thermal cycler (Applied Biosystems), programming three temperatures: at 16 °C for 30 min, 42 °C for 30 min, and 85 °C for 5 min, plus a final step at 4 °C. The volume used in the real-time PCR reaction for each miRNA was1.33 μL of the RT reaction product, which was mixed with 10 μL of TaqMan master mix (Universal PCR Master Mix, No. 4 AmpErase UNG, 2X), plus 7.67 μL of RNAse-free water and 1.0 μL of PCR probe (specific for each miRNA), giving a total of 20 μL. The analysis was performed on an Applied Biosystems QuantStudio 3 Real-Time PCR. The reaction was carried out by programming a step at 95 °C for 10 min; followed by 45 cycles of two temperatures: 95 °C for 15 s and 60 °C for 1 min. The results were analyzed by the 2^−∆∆Ct^ method, as described above in the PCR Array Analysis section.

### 2.9. Expression Analysis of miR-182-5p Target Genes via RT-qPCR

A “High Capacity cDNA Reverse Transcription Kit” (Thermo Fisher Scientific, Loughborough, UK) was used for the reverse transcription reaction according to the manufacturer’s instructions. FOXF2 and MTSS1 expression was determined by QRT-PCR using SYBR Green/ROX Master Mix (Thermo Fisher Scientific, UK) following the manufacturer’s instructions. Quantitative data were normalized relative to HPRT. The sequences of the primers were: FOXF2-For: CAA GGT AGC GTT CCC CAA TC; FOXF2-Rev: GTC TGC TTT TTT CAC ACC CTG AT; MTSS1-For: TGA CCC GCT CTG TTG; MTSS1-Rev: GGT GCC CAC TAC GGA AAC G; HPRT-For AGG GTG TTT ATT CCT CAT GG; HPRT-Rev CAC AGA GGG CTA CAA TGT. The reactions were performed in a QuantStudio 3 (Thermo Fisher Scientific, UK). The cycling conditions were: 50 °C for 2 min to activate the UNG, initial denaturation at 95 °C for 10 min, 45 cycles at 95 °C for 15 s, and finally, 60 °C for 1 min. Standard curves were analyzed to verify the amplification efficiency of each gene. The 2^−ΔΔCt^ equation was applied to calculate the relative expression in the samples.

### 2.10. Western Blot

Cells were collected and subsequently lysed using RIPA buffer (Beyotime Biotechnology Co., Ltd., Shanghai, China). The protein concentration was then determined via the Lowry method, and 50 μg was loaded onto 10% SDS-PAGE gels. After blocking, we used specific antibodies against p53 (DO-1, Santa Cruz, cat # sc-126 HRP) (1:500) and β-actin (AC-15, Sigma Aldrich, cat # A3854) (1:50,000). Protein expression was detected by chemoluminescence using Supersignal West Pico (Thermo Scientific, Waltham, MA, USA). 

### 2.11. Cell Migration and Invasion Assays

First, the cells were synchronized in G0/G1 phase by serum starvation by incubating for 24 h prior to the assay in serum-free DMEM. For all cell migration assays, the cell lines (2 × 10^5^) were resuspended in 300 μL of serum-free DMEM and seeded on the top surface of Transwell^®^ inserts (8 μm; Corning, NY, USA) in 24-well plates, to which 500 μL of 10% SFB (chemoattractant) in DMEM had previously been added. Cell invasion was assessed using a QCM ECMatrix Cell Invasion Assay in 24-well plates (8 μm ECM554 Chemicon, Millipore, Billerica, MA, USA). Cells were seeded in ECMatrix Cell Invasion Assay QCM chambers (1 × 10^5^) in the absence of fetal bovine serum and then the inserts were placed in 24-well plates to which 500 μL of 10% SFB (chemoattractant) DMEM had previously been added. Subsequently, cells were incubated at 37 °C for 24 h for the cell migration and invasion assays. Then, cells on top of the insert were gently removed, and cells that migrated to the bottom of the insert were fixed with 4% paraformaldehyde for 15 min and then stained with 0.1% crystal violet (Sigma-Aldrich, St. Louis, MO, USA). The absorbance of the stained cells was read on an ELISA plate reader (SofMaxPro, San Jose, CA, USA) at 560 nm.

### 2.12. Statistical Analysis

The statistics used were ANOVA to observe the differences between groups of different treatments and incubation times, and the unpaired Student’s *t*-test to compare the differences between two groups, using the statistical program GraphPad Prism software 5.0 (GraphPad Software, Inc., San Diego, CA, USA). Each experiment was performed in triplicate and repeated at least three times; differences were considered significant when *p* < 0.05.

## 3. Results

### 3.1. Global Expression Profiling of miRNAs in Saos-2 Cells Expressing p53R175H, p53R273C, or p53R248Q, as Well as Signaling Pathway Enrichment (KEGG) Analysis

**Figure 1:**
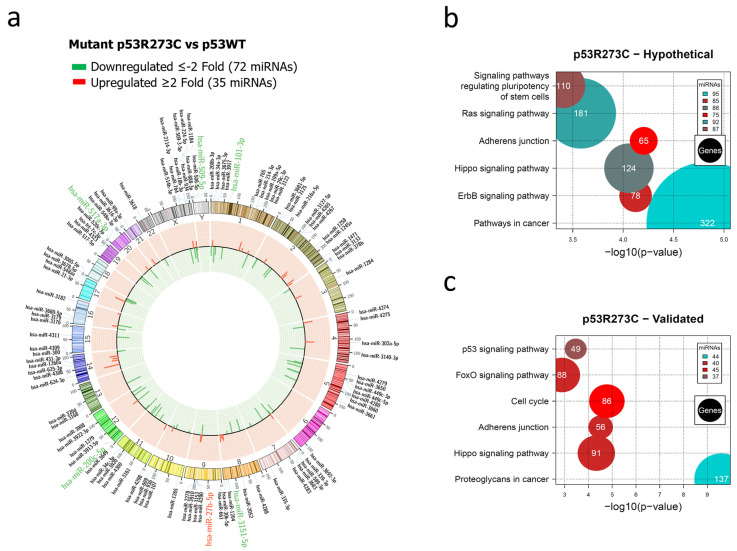
Representation of the p53R273C-regulated miRNome and signaling pathway enrichment (KEGG) analysis. (**a**) Saos-2 cells were transfected with the p53R273C mutant, and p53WT was used as a control. To determine which miRNAs were differentially expressed, a *p*-value < 0.05 and Fold Change ≥2 or ≤−2 were used. The Circos map distributes the differentially expressed miRNAs according to their chromosomal locations, within which “bar graphs” correspond to the Fold Change value of the miRNAs that were increased (red) or decreased (green) in the presence of the p53R273C mutant vs. p53WT. Additionally, the names of those miRNAs that were selected for validation through TaqMan probe assays are highlighted in red-augmented (miR-27b-5p) and green-decreased (miR-509-5p, miR-101-3p, miR-517a-3p, and miR-200c-5p). (**b**) “KEGG” pathway enrichment analysis of hypothetical target genes, based on miRPath (the DIANA-microT-CDS algorithm). (**c**) “KEGG” pathway enrichment analysis of experimentally validated genes, based on miRPath (the DIANA-microT-CDS algorithm). The scatter plot in (**b**,**c**) shows the significance level of each signaling pathway on the “X” axis (*p* < 0.05) and the pathway name on the “Y” axis. The color of the circles represents the number of miRNAs involved in the signaling pathways, and the size of the circles indicates the number of hypothetical and/or validated genes.

**Figure 2:**
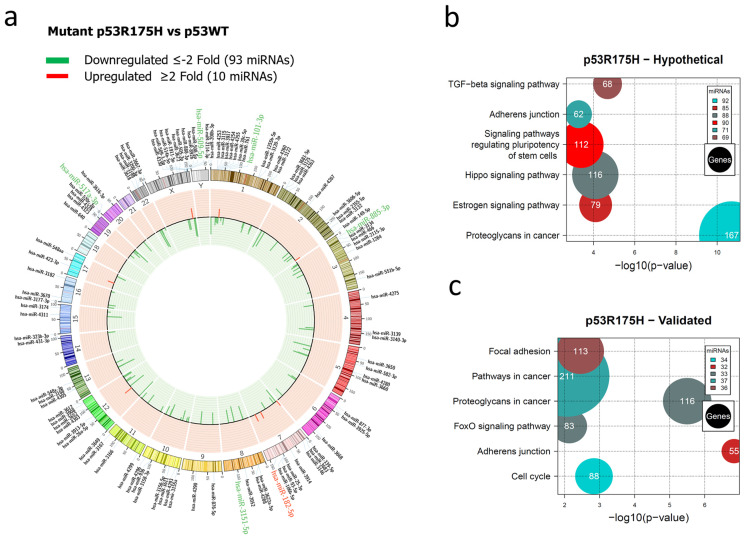
Representation of the p53R175H-regulated miRNome and signaling pathway enrichment (KEGG) analysis. (**a**) Saos-2 cells were transfected with p53R175H, and p53WT was used as a control. To determine which miRNAs were differentially expressed, a value of *p* < 0.05 and Fold Change ≥2 or ≤−2 was used. The Circos map distributes the differentially expressed miRNAs according to their chromosomal locations, within which “bar graphs” correspond to the Fold Change value of miRNAs that are increased (red) or decreased (green) in the presence of the p53R175H mutant vs. p53WT. Additionally, the names of the miRNAs that were selected for validation through TaqMan probe assays are highlighted in red-increased (miR-182-5p) and green-decreased (miR-509-5p, miR-101-3p, miR-517a-3p, miR-885-3p, and miR-3151-5p). (**b**) “KEGG” pathway enrichment analysis of hypothetical target genes, based on miRPath (the DIANA-microT-CDS algorithm). (**c**) “KEGG” pathway enrichment analysis of experimentally validated genes, based on miRPath (the DIANA-microT-CDS algorithm). The scatter plot in (**b**,**c**) shows the significance level of each signaling pathway on the “X” axis (*p* < 0.05) and the pathway name on the “Y” axis. The color of the circles represents the number of miRNAs involved in the signaling pathways, and the size of the circles indicates the number of hypothetical and/or validated genes.

**Figure 3:**
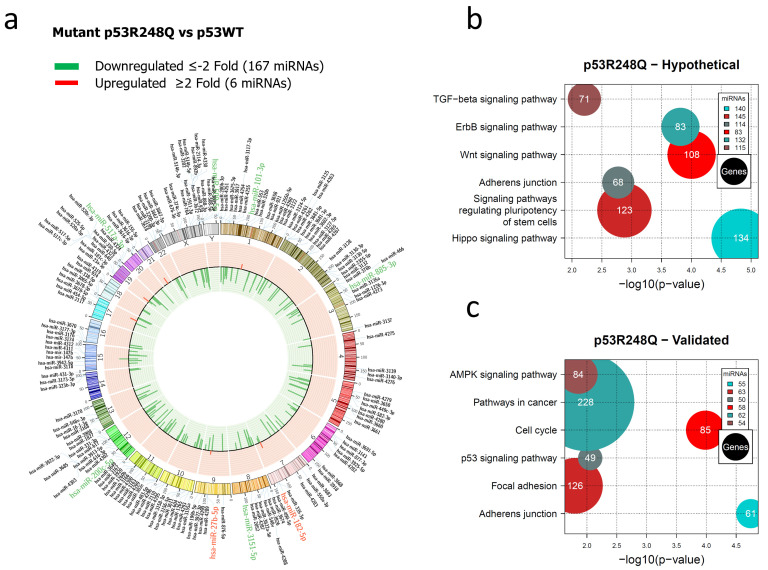
Representation of the p53R248Q-regulated miRNome and signaling pathway enrichment (KEGG) analysis. (**a**) Saos-2 cells were transfected with p53R248Q, and p53WT was used as a control. To determine which miRNAs were differentially expressed, a value of *p* < 0.05 and Fold Change ≥2 or ≤−2 was used. The Circos map distributes the differentially expressed miRNAs according to their chromosomal locations, within which “bar graphs” correspond to the Fold Change value of miRNAs that increased (red) or decreased (green) in the presence of the p53R248Q mutant vs. p53WT. Additionally, the names of the miRNAs that were selected for validation through TaqMan probe assays are highlighted in red-increased (miR-182-5p and miR-27b-5p) and green-decreased (miR-509-5p, miR-101-3p, miR-517a-3p, miR-885-3p, miR-3151-5p, and miR-200c-5p) (**b**). “KEGG” pathway enrichment analysis of hypothetical target genes, based on miRPath (the DIANA-microT-CDS algorithm). (**c**) “KEGG” pathway enrichment analysis of experimentally validated genes, based on miRPath (the DIANA-microT-CDS algorithm). The scatter plot in (**b**,**c**) shows the significance level of each signaling pathway on the “X” axis (*p* < 0.05) and the pathway name on the “Y” axis. The color of the circles represents the number of miRNAs involved in signaling pathways, and the size of the circles indicates the number of hypothetical and/or validated genes.

In order to identify novel miRNAs regulated by the most frequent p53 mutants, we performed PCR arrays of the Saos-2 cell line transfected with p53WT or with each of the p53R175H, p53R273C, and p53R248Q mutants. The p53 levels were verified by western blot (Supplementary Figure S1). Cells transfected with p53WT were used as a control, and each experimental group had three biological replicates. In addition, to determine which miRNAs were differentially expressed, cutoff points ≥2 or ≤−2 Fold Change (FC) and a value of *p* < 0.05 were used (Figure 1, Figure 2 and Figure 3).

In this study, we observed that the p53R273C, p53R248Q, and p53R175H mutants had an overall negative effect on the expression of miRNAs in cancer. The complete lists showing the miRNAs differentially expressed in the presence of p53R175H, p53R273C, or p53R248Q are in Supplementary Tables S1–S3. 

The global expression profile of the human miRNome regulated by the p53R273C mutant was represented by a Circos plot in which 72 underexpressed (green) and 35 overexpressed (red) miRNAs are visible (Figure 1a). Pathways in cancer (95 miRNAs/322 genes) presented a higher percentage of messenger RNAs (hypothetical) compared to other signaling pathways, including Adherens junction (75 miRNAs/65 genes) (Figure 1b, Supplementary Table S4). We found that these signaling pathways share 75 miRNAs. Interestingly, the Cancer pathway has 21 unique miRNAs, and Adherens junction shares almost all of them with this pathway, except for miR-3186-5p. 

In the analysis of experimentally validated target mRNAs, we found a significant enrichment of these pathways: Cancer proteoglycans (44 miRNAs/137 genes), Adherens junction (40 miRNAs/56 genes), and Cell cycle (45 miRNAs/86 genes) (Figure 1c, Supplementary Table S5). Interestingly, the most miRNAs regulating hypothetical and validated target genes belong to the Hippo pathway and Adherens junction, with 31 miRNAs in common.

For the p53R175H miRNAs profile, our results showed the downregulation of 93 and upregulation of 10 miRNAs (Figure 2a, Supplementary Table S2). In this case, hypothetical target gene analysis indicated that the Hippo signaling pathway (88 miRNAs/116 genes), the signaling pathway regulating Stem cell pluripotency (90 miRNAs/112 genes), and Adherens junction (71 miRNAs/62 genes) are shared with the miRNA profile of the p53R273C mutant. However, the Estrogen signaling pathway (85 miRNAs/79 genes) and Cancer proteoglycans (92 miRNAs/167 genes) were found exclusively in the p53R175H profile (Figure 2b, Supplementary Table S6). 

In the pathway analysis of validated target genes, we found miRNAs involved in Adherens junction (32 miRNAs/55 genes), the FoxO signaling pathway (33 miRNAs/83 genes), Proteoglycans in cancer (33 miRNAs/116 genes), Pathways in cancer (37 miRNAs/211 genes), Focal adhesion (36 miRNAs/113 genes), and Cell cycle (34 miRNAs/88 genes). Similar to the p53R273C miRNAs profile, our results revealed that “Pathways in cancer” was one of the pathways with a higher number of miRNAs and genes (Figure 2c, Supplementary Table S7).

In the miRNAs expression profile regulated by the p53R248Q mutant, we found 167 decreased and 6 increased miRNAs versus p53WT (Figure 3a, Supplementary Table S3). Furthermore, the analysis of signaling pathways regulated by hypothetical target genes showed the Hippo signaling pathway (140 miRNAs/134 genes), the signaling pathway regulating Stem cell pluripotency (145 miRNAs/123 genes), the Wnt signaling pathway (83 miRNAs/108 genes), the TGF-beta signaling pathway (115 miRNAs/71 genes), Adherens junction (114 miRNAs/68 genes), and the ErbB signaling pathway (132 miRNAs/83 genes) (Figure 3b, Supplementary Table S8). 

Notably, the p53R273C, p53R175H, and p53R248Q miRNA profiles share the Hippo signaling pathway, the pathway regulating Stem cell pluripotency, and Adherens junction. 

To further investigate the mechanisms by which mutant p53R248Q contributes to cancer development, we performed an analysis of validated target genes using the miRPath algorithm, which identified multiple genes involved, especially in pathways such as Cell cycle (58 miRNAs/85 genes), Adherens junction (55 miRNAs/61 genes), Pathways in cancer (62 miRNAs/228 genes), p53 signaling pathway (50 miRNAs/49 genes), MAPK signaling pathway (54 miRNAs/84 genes), and Focal adhesion (63 miRNAs/126 genes) (Figure 3c, Supplementary Table S9). 

Remarkably, the “Cell cycle” pathway is shared among the three expression profiles. Specifically, we found the miRNAs miR-200b-3p, miR-431-3p, miR-508-5p, miR-509-5p, miR-520c-3p, miR-888-3p, miR-3140-3p, miR-3913-5p, and miR-101-3p have been reported and experimentally validated to be involved in the cell cycle, and they are shared among p53R175H, p53R248Q, and p53R273C. Finally, it is important to note that the three mutant profiles also share miRNAs (miR-200b-3p, miR-3140-3p, miR-508-5p, miR-509-5p, miR-888-3p, and miR-101-3p) involved in the regulation of the Adherens junction pathway in both hypothetical and validated target analyses. These findings give us insight into the functions in which p53 mutants might be involved through the regulation of miRNAs in cancer.

### 3.2. Heat Map and Venn Diagram of Differentially Expressed miRNAs in p53R273C, p53R248Q, and p53R175H Mutants

**Figure 4:**
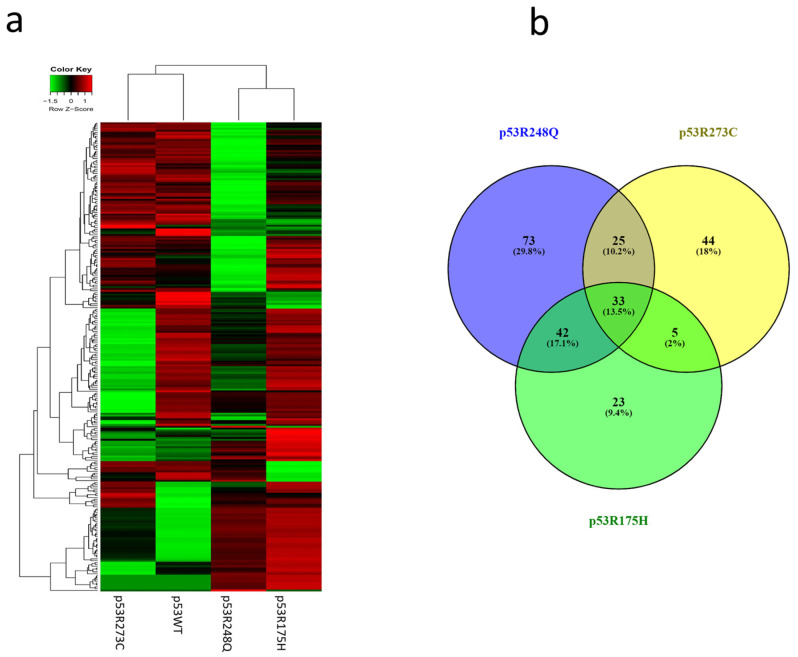
Heat map and Venn diagram of differentially expressed miRNAs in Saos-2 cells transfected with p53R273C, p53R248Q, and p53R175H mutants. (**a**) The Heat map represents the color-coded expression levels: red indicates overexpression, and green indicates underexpression. The expression of each miRNA is hierarchically grouped on the “Y” axis, and the p53 mutants are represented on the “X” axis. (**b**) The Venn diagram shows the overlap of miRNAs among the three p53 mutants (33 miRNAs). The p53R273C mutant shares 25 miRNAs with p53R248Q and only 5 with p53R175H. In addition, the p53R248Q mutant exclusively regulates 73 miRNAs, while 42 are shared with the p53R175H mutant.

After analyzing the signaling pathways of each expression profile, we performed hierarchical clustering of the miRNAs. The results in the Heat map show that the expression profile of p53WT has a similar pattern to the p53R273C and p53R248Q mutants but is different from p53R175H (Figure 4a). This is also reflected in the Venn diagram, where the p53R273C mutant shares 25 miRNAs with p53R248Q and only 5 with p53R175H (Figure 4b). In addition, the p53R248Q mutant shares 42 miRNAs with the p53R175H mutant, while the p53R248Q mutant exclusively regulates 73 miRNAs (Figure 4b). Interestingly, the p53R175H mutant shows fewer shared miRNAs, but its expression profile is similar to p53R248Q (Figure 4a). Moreover, it is worth mentioning that the three mutants have 33 miRNAs in common, and 72 others are shared only by two mutants in any of their combinations (Figure 4b).

### 3.3. Validation of Differentially Expressed miRNAs in the Presence of the p53R273C, p53R248Q, and p53R175H Mutants

**Figure 5:**
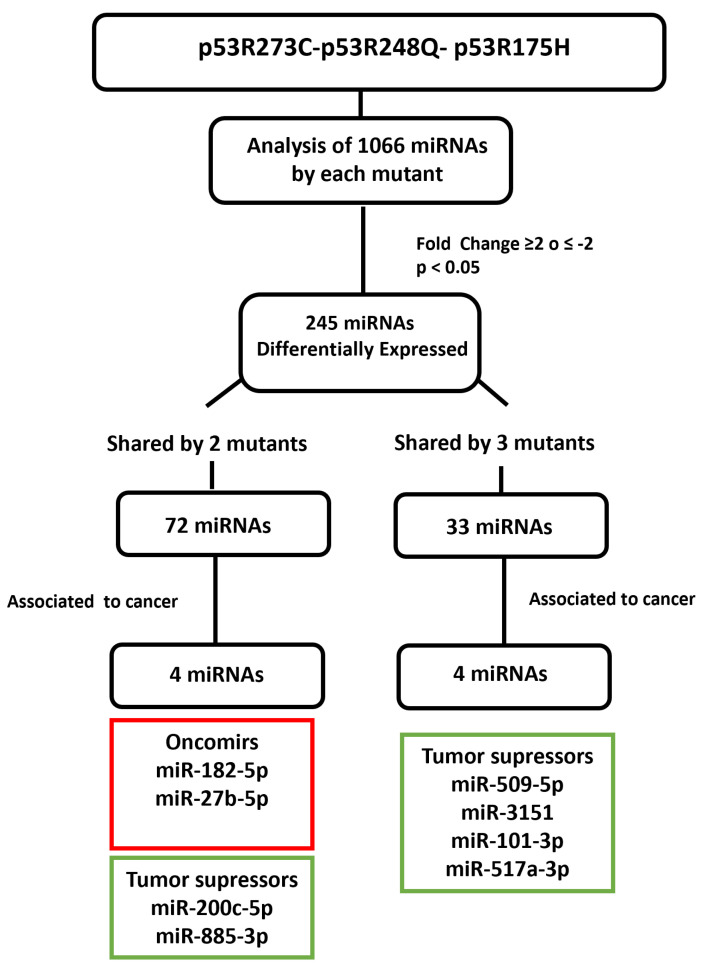
Flowchart showing the selection mechanism of miRNAs validated by Taqman probes assays. PCR arrays (miRBase version 16, 1066 miRNAs; Qiagen) of the mutants (p53R175H, p53R273C, and p53R248Q) were performed and 254 differentially expressed miRNAs were identified according to cutoff points of ≥2 or ≤−2 Fold Change and a value of *p* < 0.05. Subsequently, we found that 72 miRNAs were shared by at least two p53 mutants and 33 by all three. We also identified these miRNAs’ association with cancer through a literature survey. With this strategy, we selected and validated the downregulation of six tumor suppressor miRNAs: miR-509-5p, miR-200c-5p, miR-3151-5p, miR-885-3p, miR-517a-3p, and miR-101-3p, as well as two oncomiRs: miR-182-5p and miR-27b-5p.

After determining which miRNAs were differentially expressed in the presence of the p53R273C, p53R248Q, and p53R175H mutants, we selected some of them to validate our results. We obtained 245 differentially expressed miRNAs using a cutoff of ≥2 or ≤−2 Fold Change and a *p* < 0.05; 72 miRNAs were shared by at least two mutants and only 33 by all three mutants. Then, we searched in the literature for an association between the miRNAs differentially expressed in response to at least two p53 mutants. With this strategy, we selected and validated the downregulation of six tumor suppressor miRNAs: miR-509-5p [25,26], miR-200c-5p [27,28], miR-3151-5p [29], miR-885-3p [30,31,32], miR-517a-3p [33,34,35], and miR-101-3p [36,37,38], as well as two oncomiRs: miR-182-5p [39,40,41,42,43,44] and miR-27b-5p [45,46,47] (Figure 5).

**Figure 6:**
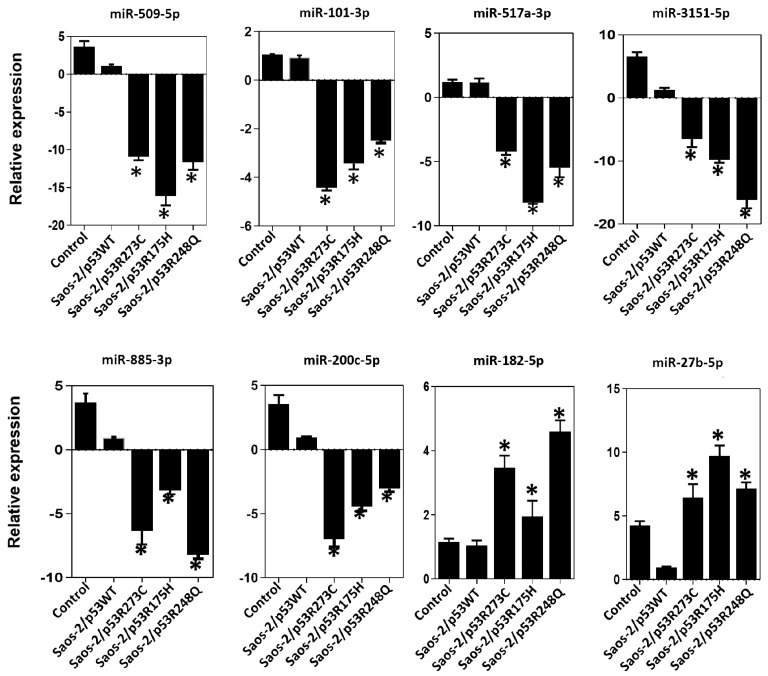
Validation of miRNAs by TaqMan probe assays. Relative expression of miR-509-5p, miR-101-3p, miR-517a-3p, miR-3151-5p, miR-885-3p, miR-200c-5p, miR-182-5p and miR-27b-5p in the cell line Saos-2 transfected with empty vector (Control) or with mutant p53 (Saos-2/p53R248Q, Saos-2/p53R273C or Saos-2/p53R175H) vs. Saos-2/p53WT. Data are presented as the two-fold change in miRNA level normalized to U6 (endogenous control). Data represent mean ± SD (n = 3), and (*) refers to expression changes that are significant compared to cells transfected with p53WT (Saos-2/p53WT) (*p* < 0.05).

In the expression profile of the p53R273C mutant, we observed decreased expression of tumor suppressors miR-509-5p (~−2.77 fold), miR-101-3p (~−48.74 fold), miR-517a-3p (~−91.74 fold), miR-3151-5p (~−2.56 fold), and miR-200c-5p (~−5.38 fold), and increased of the oncomiR miR-27-5p (~2.66 fold) (Figure 1a, Supplementary Table S1). To validate these results, we performed real-time qRT-PCR with TaqMan probes, and the same tendency was obtained for miR-509-5p (~−10.93 fold), miR-101-3p (~−4.41 fold), miR-517a-3p (~−4.22 fold), miR-3151-5p (~−6.51 fold), miR-200c-5p (~−6.98 fold), and miR-27-5p (~−6.25 fold) (Figure 6). Notably, in the validations with TaqMan probes, we also found decreased miR-885-3p (~−6.35 fold) and an increase in miR-182-5p (~3.46 fold), which were not differentially expressed in the miRNA expression profile (Figure 1a and Figure 6). 

Similarly, the p53R175H mutant miRNA profiling analysis showed negative regulation of miR-509-5p (~−54.42 fold), miR-101-3p (~−7.27 fold), miR-517a-3p (~−67.41 fold), miR-3151-5p (~−47.37 fold), and miR-885-3p (~−4.71 fold); but positive regulation of miR-182-5p (~8.54 fold) (Figure 2a, Supplementary Table S2). These miRNAs were validated by real-time PCR with Taqman probes, and the results were consistent with the miRNA profiling analysis (miR-509-5p/~−16.12 fold), (miR-101-3p/~−3.42 fold; miR-517a-3p/~−8.17 fold; miR-3151-5p/~−9.82 fold); miR-885-3p/~−3.20 fold); miR-182-5p/~1.95 fold) (Figure 2a and Figure 6). Additionally, in real-time validation with Taqman probes, we also found a decrease in miR-200c-5p expression (~−4.98 fold) and an increase in miR-27-5p (~9.95 fold) (Figure 6). 

Finally, in the p53R248Q mutant expression profile of miRNAs, we observed the downregulation of miRNA tumor suppressors miR-509-5p (~−11. 99 fold), (miR-101-3p (~−73.98 fold), miR-517a-3p (~−845.16 fold), miR-3151-5p (~−10.27 fold), miR-885-3p (~−43.98 fold), and miR-200c-5p (~−3.57 fold); in contrast, miR-182-5p was positively regulated (~6.87 fold) (Figure 3a, Supplementary Table S3). Validation by qRT-PCR showed the following results: miR-509-5p (~−11.63 fold), miR-101-3p (~−2.47 fold), (miR-517a-3p (~−5.47 fold), miR-3151-5p (~−16.15 fold), miR-885-3p (~−8.22 fold), miR -200c-5p (~−3.04 fold), and miR-27-5-p (~6.86 fold) (Figure 6). The qRT-PCR analysis with TaqMan probes showed overexpression of miR-182-5p in the presence of all three mutants (Figure 6).

### 3.4. Mutant p53R248Q Is Associated with Overexpression of miR-185-5p and Downregulation of FOXF2 and MTSS1

**Figure 7:**
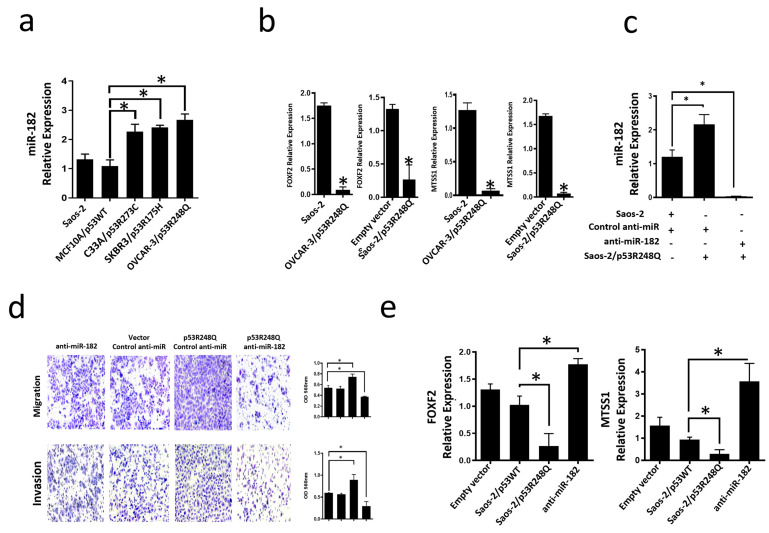
Mutant p53R248Q stimulates invasion and migration through miR-182 upregulation in Saos-2 cells. (**a**) Relative expression of miR-182-5p in Saos-2 cell line (null p53) and cell lines with endogenous mutant p53 (OVCAR-3/p53R248Q, C33a/p53R273C and SKBR3/p53R175H) vs. MCF10a/p53WT. (**b**) Relative expression of FOXF2 and MTSS1 in Saos-2 (null p53) vs. OVCAR-3/p53R248Q (endogenous p53R248Q) and in Saos-2 transfected with empty vector (Empty vector) vs. Saos-2/p53R248Q (transfected with p53R248Q). (**c**) Relative expression of miR-182-5p in Saos-2 cells transfected with Control-anti-miR/Vector; Saos-2 transfected with p53R248Q and control anti-miR (p53R248Q/control-anti-miR) or transfected with antimiR-182 (p53R248Q/antimiR-182). RT-PCRs were normalized with GAPDH and/or U6, respectively. (**d**) Cell invasion and migration assays of Saos-2 cells transfected with Control-anti-miR/Vector; Saos-2 transfected with p53R248Q and control anti-miR (p53R248Q/control-anti-miR) or transfected with antimiR-182 (p53R248Q/antimiR-182). (**e**) Relative expression of FOXF2 and MTSS1 in Saos-2 cells transfected with empty vector (Empty vector), transfected with p53WT (Saos-2/p53WT), transfected with p53R248Q and control anti-miR (Saos-2/p53R248Q) or transfected with antimiR-182 (antimiR-182). Error bars represent (mean ± SD) from three independent experiments (n = 3), * *p* < 0.05.

miR-182-5p is an oncomiR that is overexpressed in several cancer types; it has been reported to inhibit the expression of targets such as FOXF2 (Forkhead box F2) and MTSS1 (Metastasis suppressor-1). To determine whether the endogenous presence of the three most frequent p53 mutants could be associated with increased miR-182-5p expression, we performed qRT-PCR assays on cell lines expressing these mutants (OVCAR-3/p53R248Q, C33a/p53R273C, and SKBR3/p53R175H). Our results showed that the three cell lines endogenously expressing the three mutants significantly overexpress miR-182-5p compared to the cell line MCF10a (p53WT) or Saos-2 (null p53) (Figure 7a). In contrast, the miR-185-5p target genes, FOXF2 and MTSS1, exhibited significantly low expression in the Saos-2-p53R248Q and OVCAR-3 cell lines compared with their respective controls (Saos2 and Saos-2 transfected with an empty plasmid) (Figure 7b), which is consistent with the overexpression of miR-182-5p in these cell lines. 

### 3.5. The p53R248Q Mutant Stimulates Invasion and Migration by Overexpressing miR-182-5p in Saos-2 Cells

To assess whether the induction of migration and invasion by the p53R248Q mutant depends on miR-182-5p expression, we transfected the Saos-2 cell line with control-anti-miR (scramble control), transfected with p53R248Q and control-anti-miR, or transfected with p53R248Q and antimiR-182. First, we evaluated the inhibition of the expression of miR-182-5p by the siRNAs (Figure 7c), where we observed a decrease of mir-182 in the presence of the anti-miR-182 in cells transfected with the p53R248Q mutant and then evaluated their effect on cell migration and invasion (Figure 7d). We found that inhibition of miR-182-5p by shRNAs significantly decreased the migration and invasion abilities of the Saos-2 cell line transfected with the p53R248Q mutant compared with scramble-transfected control cells (Figure 7d). Furthermore, we observed that inhibiting miR-182-5p in the presence of the p53R248Q mutant restored the expression of MTSS1 and FOXF2 (Figure 7e), which is related to the decreased effect on migration and invasion mentioned above (Figure 7d). Our results suggest that the p53R248Q mutant depends on miR-182-5p to induce cell migration and invasion in the Saos-2 cell line. 

### 3.6. Inhibition of the p53R248Q Mutant in the OVCAR-3 Cell Line Induces Decreased Cell Migration and Invasion through miR-182-5p

**Figure 8:**
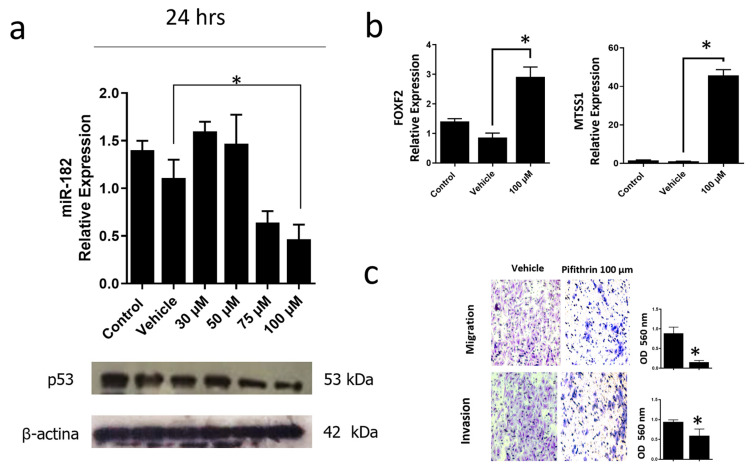
Inhibition of mutant p53R248Q in OVCAR-3 cells decreases cell invasion and migration through miR-182. (**a**) Relative expression of miR-182-5p and western blot of p53R248Q in the cell line OVCAR-3 (endogenous p53R248Q) treated with pifithrin-α (30 μM, 50 μM, 75 μM, or 100 μM) for 24 h, using as controls cells without treatment (Control) and treated only with vehicle (Vehicle). (**b**) Relative expression levels of FOXF2 and MTSS1 in OVCAR-3 cells (endogenous p53R248Q) treated with pifithrin-α for 24 h compared with cells without treatment (Control) and only vehicle (Vehicle). (**c**) Invasion and migration assays of OVCAR-3 cells with vehicle (Vehicle) or treated with pifithrin-α (Pifithrin 100 μM). Error bars represent mean ± SD of three independent experiments (n = 3), * *p* < 0.05.

To corroborate that the p53R248Q mutant induces overexpression of miR-182-5p, we inhibited the expression of this mutant in the OVCAR-3/p53R248Q cell line with pifithrin, a p53 inhibitor. First, we performed a dose-response curve of pifithrin of 30, 50, 75, and 100 μM and observed a decrease of p53R248Q protein at 75 and 100 μM concentrations. Subsequently, we selected the concentration (100 μM) where we observed a more significant effect on p53 knockdown and analyzed the expression of miR-182-5p. Our results showed that miR-182-5p significantly decreased with pifithrin treatment, coinciding with a decrease in the expression of the p53R248Q mutant (Figure 8a). 

In addition, we also evaluated the miR-182-5p target genes (FOXF2 and MTSS1) and observed an increase in FOXF2 and MTSS1 expression in pifithrin-treated cells (Figure 8b). 

Finally, we performed cell migration and invasion assays. Our results showed that pifithrin treatment decreased the ability of OVCAR3 cells to invade or migrate compared with vehicle-treated control cells (Figure 8c). These results suggest that the p53R248Q mutant positively regulates miR-182-5p expression, which results in the downregulation of FOXF2 and MTSS1, and this is associated with an increase in cell migration and invasion.

## 4. Discussion

In this study, we observed that most of the differentially expressed miRNAs were downregulated in the presence of p53R175H, p53R273C, and p53R248Q. Our results agree with a previous study in which an miRNA profile was obtained in the presence of the p53R282W mutant: they also observed an overall negative effect on miRNA expression [48]. In addition, Garibaldi et al. demonstrated that mutants bind and sequester the p72/82 RNA helicase of the microprocessor complex, interfering with the association between Drosha and pri-miRNA, inhibiting post-transcriptional maturation, which contributes to the negative regulation of miRNAs [35]. 

The association between the differentially expressed miRNAs in the three miRNAs profiles and the signaling pathways in which they might participate (target mRNAs hypothetical/mRNAs validated) led us to conclude that they have the “Adherens junction” pathway in common. This coincides with reports that p53 mutants can promote a mesenchymal phenotype, inducing transcription factors such as TWIST-1 and SLUG, which promotes the loss of adherens junction, favoring cell motility [49]. In addition, the dysregulation of some miRNAs involved in the regulation of EMT, metastasis, cell migration, and invasion (miR-130b, let-7i, miR-218, and miR-519a) has been previously reported in the presence of p53 mutants [13]. 

In another study, the exogenous expression of p53R248Q and p53R282W mutants in the H1299 cell line (null p53) drove invasion through miR-155 overexpression in breast cancer. Moreover, knockdown of the R249S endogenous p53 mutant in BT-549 cells resulted in a significantly reduced level of miR-155, confirming a role for mutant p53 in the aberrant activation of miR-155 [50].

In agreement with these reports, we also observed overexpression of miR-155 in the presence of the p53R248Q mutant. However, we also observed different pathways altered depending on which p53 mutant was expressed. This is in agreement with studies of GOF activities of p53 mutants. For example, in mice harboring a novel germline Trp53R245W allele (contact mutation), compared with mice with Trp53R172H (structural mutation) and Trp53R270H (contact mutation) alleles, it was observed that Trp53R245W/+ and Trp53R270H/+ mice developed osteosarcomas more frequently and had a poor overall survival in contrast with Trp53R172H/+ mice [11].

Previous research showed that p53R248Q and p53R248W mutants induce invasion and migration by binding to Stat3 and enhancing the activation of Stat3 phosphorylation in colorectal and pancreatic cancer [51,52]. Interestingly, a positive correlation has been reported between miR-182-5p and the Stat3 pathway in gliomas and breast cancer [42,53]. Recently, it has also been demonstrated that miR-182-5p targeted PIAS1 (protein inhibitor of activated STAT) mRNA in endometrial cancer, and the overexpression of PIAS1 inhibited Stat3 activity [43]. These reports suggest that p53 mutants could induce activation of the Stat3 pathway through miR-182-5p in addition to binding to the protein directly; however, more experiments are necessary to prove this hypothesis. 

Other signaling pathways shared in the three profiles based on target hypothetical mRNAs were the Hippo signaling pathway and the pluripotent Stem cell regulation pathway, which in turn share several miRNAs (miR-101-3p, miR-3125, miR-3681-5p, miR-508-5p, miR-517a-3p, miR-888-3p, miR-200b-3p, miR-2052, miR-3122, miR-3140-3p, miR- 3151-5p, miR-3168, miR-3618, miR-3660, miR-3908, miR-3913-5p, miR-4267, miR-4275, miR-4280, miR-4288, miR-4311, miR-4323, miR-509-5p and miR-520c-3p). The Hippo signaling pathway is a key regulator of physiological processes such as cell proliferation, differentiation, polarity, and death [54,55]. Previously, it was reported that the p53R280K and p53R175H mutants physically interact with a modulator of this pathway known as YAP (YES-associated protein) and form a complex with NF-Y, increasing the transcription of genes involved in cell proliferation [56]. In addition, YAP/TAZ overexpression has been reported to induce cell proliferation and the acquisition of cancer stem cell characteristics [57]. It could be possible that p53R248Q, p53R175H, and p53R273C regulate the expression of some miRNAs through the Hippo pathway or induce this pathway by regulating some of these miRNAs; it would be interesting to analyze this possibility. 

Moreover, analysis through the DIANA-TarBase (validated mRNAs) showed that the Cell cycle is a shared pathway among the three miRNA expression profiles from the three p53 mutants, with the following miRNAs in common: miR-200b-3p, miR-431-3p, miR-508-5p, miR-509-5p, miR-520c-3p, miR-888-3p, miR-3140-3p, and miR-3913-5p. It is well known that activation of p53WT by DNA damage induces the expression of p21. However, the above does not occur in cells expressing p53 mutants, as p21 expression decreases [58]. Furthermore, it has been reported that p53 mutants can regulate the cell cycle through miR-128-2, miR-223, and miR-517a [13]. 

Interestingly, in our work, we found downregulation of miR-517a-3p, miR-509-5p, and miR-101-3p in the presence of all three mutants. In this sense, previous studies have shown that downregulation of miR-517a and miR-517c contribute to the development of hepatocellular carcinoma through post-transcriptional regulation of Pyk2 (protein tyrosine kinase 2 beta), which is associated with blockade of the G2/M transition [34]. In our study, we found decreased miR-517a and miR-517c in the profile of the p53R248Q mutant. miR-509-5p can also delay the G1/S transition in the cell cycle, as well as facilitate apoptosis in cervical cancer cells. This is because miR-509-5p negatively regulates MDM2, which increases p53WT levels, resulting in p21 overexpression [25]. 

Additionally, upregulation of miR-101-3p has been reported to suppress EZH2 and HDAC9 expression, thereby inhibiting cell cycle progression in retinoblastoma cells [59]. HDAC9 expression is associated with cell proliferation in vitro, and its inhibition with cell arrest in the G1 phase is consistent with the reduction of Cyclin E2 and CDK2 expression in retinoblastoma cells [60]. On its part, EZH2 transcriptionally represses the cell cycle suppressor INK-ARF, driving cell cycle progression of cancer stem cells [61], so p53 mutants could induce cell cycle progression by inducing Cyclin E2 and CDK2 expression and silencing INK-ARF expression through negative regulation of miR-101-3p.

In addition, it has been reported that miR-3151 is silenced by methylation of its promoter in chronic lymphocytic leukemia (CLL), favoring cell proliferation [29]. Although inactivation of this miRNA has not been associated with the presence of p53 mutations, it is known that mutations of this protein are frequent in patients with CLL and have been associated with resistance to chemotherapy and a poor prognosis [62], so it would be interesting to demonstrate whether there is an association between the presence of p53 mutants and miR-3151 expression, as well as its possible relationship with chemotherapy resistance and/or prognosis in patients with CLL. 

The miRNAs are known to repress gene expression, but WT-induced overexpression of ΔNp63α (a dominant-negative isoform of p63) in cisplatin-treated cells has been reported to activate MDM4 (MDM4 regulator of p53) expression. Upregulation of miR-885-3p promotes p53-dependent cisplatin-induced mitochondrial pathway apoptosis in WT ΔNp63α-expressing head and neck squamous carcinoma cells through overexpression of MDM4 and downregulation of BCL2 (B-cell lymphoma 2). Interestingly, a decrease of MDM4 is related to resistance to cisplatin [31]. It has also been shown that mutant p53R273H and p53WT can interact with ΔNp63α, mediating its degradation [63]. Thus, it would be interesting to demonstrate whether there is an association with cisplatin resistance through p53 mutants dependent on inhibition of the ΔNp63α protein. 

Furthermore, miR-200c-5p suppresses proliferation and metastasis, inhibiting MAD2L1 (mitotic arrest deficient 2 like 1) in hepatocellular carcinoma [27]. It should be noted that several studies have shown that p53 is frequently mutated in this type of cancer [64,65]. Furthermore, the presence of p53 mutations correlates with tumor progression and survival in hepatocellular carcinoma [66], suggesting an important role of p53 mutations in hepatocellular carcinoma. 

In addition, in our study, we observed overexpression of miR-27b-5p in the presence of p53 mutants. miR-27b has been reported as an oncomiR and a tumor suppressor, which has also been observed for other miRNAs. It has been suggested that the cellular context is important to determine the expression and function of miRNAs. The balance between the targets of each miRNA present in particular situations and specific tissues. For example, miR-27b is overexpressed in breast, gastric, ovarian, and glioma cancers, where it has been associated with the induction of processes such as cell proliferation, metabolism, migration, and invasion [45,47]. In addition, miR-27b is overexpressed in the MDA-MB-231 breast cancer cell line. These levels increase in a subline selected for its high capacity to induce metastasis to the lung, called 4175. It was also demonstrated that inhibiting miR-27b expression decreases these cells’ migration and invasion capacity. Interestingly, the MDA-MB-231 cell line has a mutation in the p53 gene, so it would be interesting to analyze whether miR-27b expression is associated with the presence of p53 mutations in breast cancer [45]. 

Finally, miR-182-5p is also considered an oncomiR because it is closely related to migration, invasion, and metastasis [39,40,41,67]. The active participation of adhesion molecules in the metastatic capacity of tumor cells is crucial since alterations of their expression generate a loss of function of the adhesion complex and gives rise to processes such as cell migration and invasion. This is consistent with the alteration of several miRNAs related to the signaling pathway “Adherens junction” in the expression profiles of p53 mutants, especially in the presence of the p53R248Q mutant. Among the differentially expressed miRNAs involved in regulating processes such as "Adherens junction" is miR-182-5p. Some of the target genes of this miRNA have already been experimentally validated, such as FOXF2 and MTSS1 [41,44,67]. FOXF2 is a negative regulator of TWIST1 (Transcriptional repressor of E-cadherin) and it also negatively regulates the expression of matrix metalloproteinases such as MMP1 [68]. MTSS1 suppresses the formation of F-actin fibers, which is an important event in the rearrangement of the cytoskeleton in cell migration and invasion processes. Besides, MTSS1 accelerates the kinetics of adherens junction assembly and makes cells more resistant to cell-cell junction disassembly [69,70]. 

In this study, we demonstrated that the three most frequent p53 mutants in cancer induce overexpression of miR-182-5p. In addition, we also observed miR-182-5p overexpression in the OVCAR-3/p53R248Q cell line, which coincides with low expression levels of the miRNA target genes FOXF2 and MTSS1. In agreement with our results, in high-grade serous ovarian carcinoma (HG-SOC), it has been reported that p53 mutations are frequent, and overexpression of miR-182 is common in the early stages [44]. Likewise, Xu et al., in 2014, observed that the OVCAR-3 cell line (p53R248Q) overexpressed miR-182-5p compared to the SKOV3 cell line (p53 null) [69]. Of note, these studies did not associate the overexpression of miR-182-5p with the presence of p53R248Q.

Additionally, we demonstrated that inhibiting miR-182-5p in the presence of mutant p53R248Q reestablished FOXF2 and MTSS1 expression, which correlated with decreased cell migration and invasion. Interestingly, the therapeutic potential of anti-miR-182 has been suggested in an orthotopic animal model to mimic human ovarian cancer, using the cell lines SKOV3 with transfection of miR-182 (intrabursal injection) and OVCAR-3 (intraperitoneal injection). In these models, they demonstrated that treatment with anti-miR-182-5p decreased tumor size, invasion, and distant metastasis compared to control [69]. 

Moreover, Wang et al., in 2017, observed that miR-182 overexpression could promote the proliferation and migration of cancer cell lines from head and neck squamous cell carcinoma (HNSCC), presenting TP53 mutations [71]. Notably, in this study, they only observed an association between miR-182-5p overexpression and the presence of p53 mutations in patients and cell lines from HNSCC and did not demonstrate whether p53 mutants induce miR-182-5p overexpression.

## 5. Conclusions

**Figure 9:**
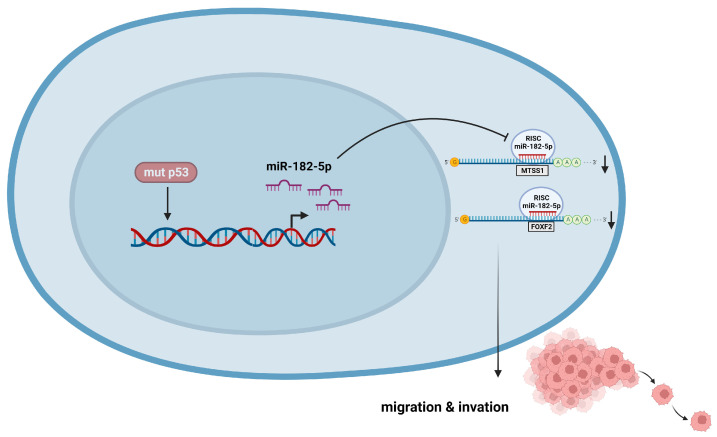
p53R248Q induces cell migration and invasion through overexpression of miR-182-5p in cancer. The p53R248Q mutant induces overexpression of miR-182-5p, which promotes downregulation of MTSS1 and FOXF2 and increases cell migration and invasion. Mutant p53R248Q is required for the upregulation of miR-182-5p because inhibition of mutant p53 by pifithrin-α has a negative effect on the expression of miR-182-5p and its targets, leading to decreased cell migration and invasion.

We found a direct relationship between the presence of p53 mutations and miR-182-5p overexpression because inhibition of the p53R248Q mutant in the OVCAR-3 cell line decreased miR-182-5p expression and correlated with decreased migration and invasiveness of OVCAR-3 cells, as well as restoration of the expression of its target genes FOXF2 and MTSS1. These results suggest that the ability of the p53R248Q mutant to induce cell migration and invasion is dependent on miR-182-5p expression (Figure 9). This is the first time that regulation of miR-182-5p expression by p53 mutants has been reported. However, further studies are needed to elucidate the mechanism by which p53 mutants induce miR-182-5p overexpression. In summary, our study provides a comprehensive overview of the regulation of miRNAs by the most frequent p53 mutants in cancer, contributing to the knowledge of how p53 mutants can induce cancer development through the regulation of miRNA expression. These results could also suggest new specific therapeutic strategies for cancer patients with p53 mutations.

## Data Availability

Not applicable.

## Supplementary Materials

The following supporting information can be downloaded at: https://www.mdpi.com/article/10.3390/cells12202506/s1.
