# Supplementary figures and images for: Mutant p53 Gain-of-Function Induces Migration and Invasion through Overexpression of miR-182-5p in Cancer Cells
Source: Cells. 2023 Oct 23;12(20):2506. doi: 10.3390/cells12202506 (PMC10605582; doi:10.3390/cells12202506)

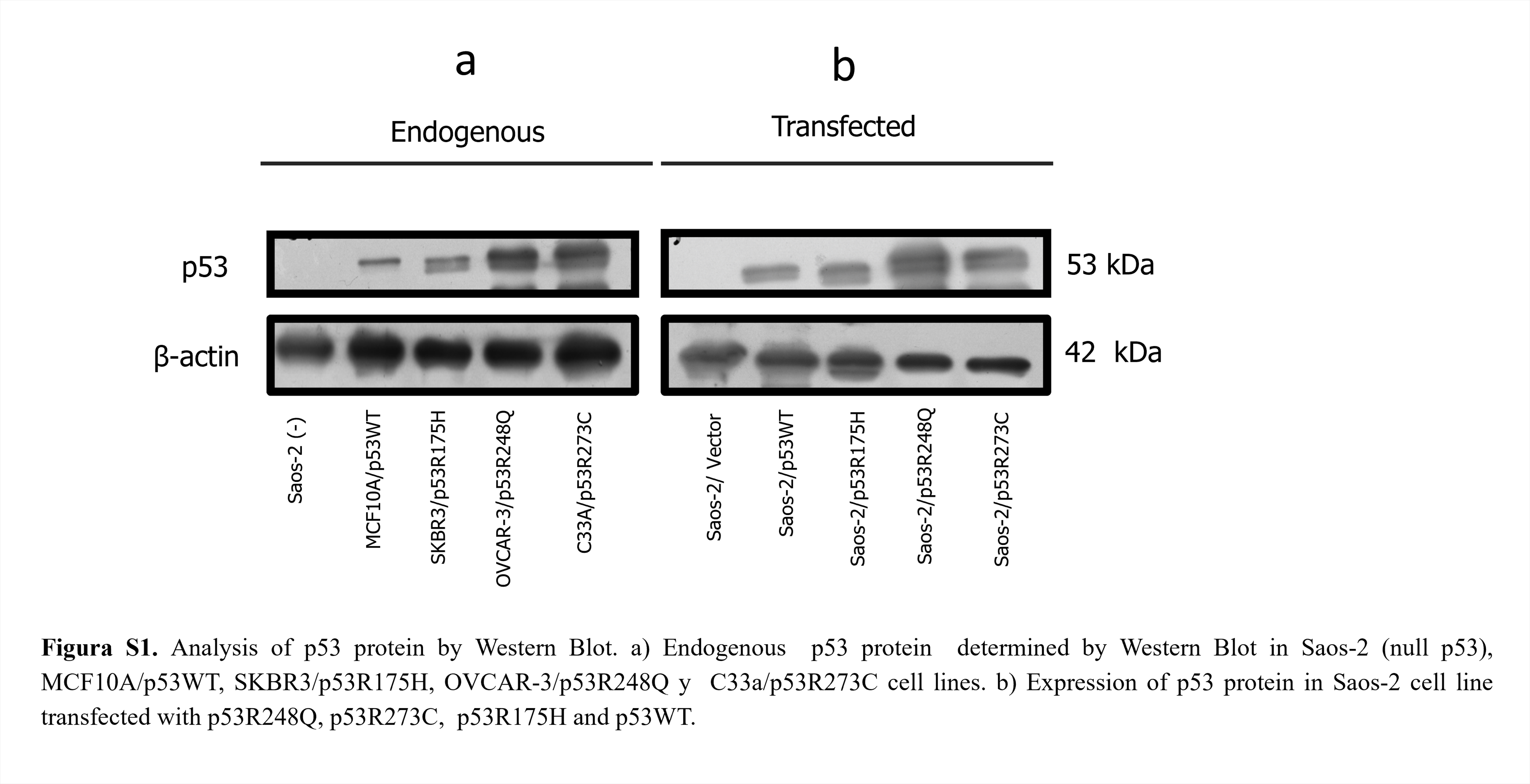

Supplement: Supplementary file 1 [file cells-12-02506-s001.zip › cells-2525754-Figure S1.tif]
